# Supplementary material for: Antibiotic usage practices and its drivers in commercial chicken production in Bangladesh
Source: PLoS One. 2022 Oct 17;17(10):e0276158. doi: 10.1371/journal.pone.0276158 (PMC9576089; doi:10.1371/journal.pone.0276158)
Supplement: S1 File — (DOCX) [file pone.0276158.s001.docx]

**Supplement 1: Questionnaire for AMU data collection in commercial poultry farm**

| 1 | Farm ID: | | | Date: |
| --- | --- | --- | --- | --- |
| 2 | Farmer’s name: | | | Address: |
| 3 | Phone number: | | |  |
| 4 | GPS (farm location): | | |  |
| 5 | Types of poultry farm:  □ Broiler  □ Layer  □ Sonali | | | |
| 6 | Number of poultry species: | | | |
| 7 | Number of poultry shed: | | | |
| 8 | Number of chicken/square feet: | | | |
| 9 | Total number of chicken:  □ Broiler:  □ Layer:  □ Sonali:  □ Backyard chicken:  □ Other poultry:______________ | | | |
| 10 | Total number of batch: | | | |
| 11 | Age of the flock  □ Batch 1:____________  □ Batch2:____________  □ Others:_____________ | | | |
| 12 | Type of poultry feed used: □Commercial □Farm made □Both | | | |
|  | If feed is prepared within farm, any antibiotic or probiotic are used in feed:  □ antibiotic □ antibiotic □ No | | | |
| 13 | Chicken morbidity (last 14 days) (no.):  Chicken mortality (last 14 days) (no.): | | | |
| 14 | Source of day old chicks:  □ Hatchery  □ Feed dealer  □ Others_________________ | | | |
| 15 | Any negotiation between you and feed dealer: □ Yes □ No  If yes, What types of support (multiple answer)  □ Supply day-old chick  □ Supply feed  □ Supply medicine  □ Credit support  □ Others_____________________ | | | |
| 16 | Where you sale your chicken products (multiple answer)?  □ Local market  □ Wholesale market  □ Feed dealer  □ Middlemen  □ Others____________________ | | | |
| Antimicrobial use data | | | | |
| 17 | What type drugs used for this farm chicken (multiple answer):  □Antibiotic  □Antiprotozoa  □Antiparasitic  □ Vitamin  □Mineral  □Growth Promoter  □ Probiotics  □ Vaccine  □Others________________ | | | |
| 18 | Antibiotics uses (**TODAY**) :  □Yes □No | If yes, mention the name of the antibiotics (generic name):  □__________________________□________________________  □__________________________□________________________ | | |
|  |  | Route of administration:  □Water □Feed □Injection □Spray□ Mixed | | |
|  |  | Purposes of antibiotic use:  □Prophylactic □Therapeutic □Both | | |
|  |  | Clinical signs (current illness): | | |
|  |  | Daily dosage:  □ Antibiotic name ________________ Dose______/100 chicken  □ Antibiotic name ________________ Dose______/100 chicken  □ Antibiotic name ________________ Dose______/100 chicken  □ Others______________ | | |
|  |  | Antibiotic use duration:  □ Antibiotic name ________________day(s)____________  □ Antibiotic name ________________ day(s)____________  □ Antibiotic name ________________ day(s)____________ | | |
| 19 | Antibiotics uses (**Last 14 days**) :  □Yes □No | If yes, mention the name of the antibiotics (generic name):  □__________________________□________________________  □__________________________□________________________ | | |
|  |  | Purposes of antibiotic use (multiple answer):  □Prophylactic  □Therapeutic  □Both | | |
| 20 | Timing of antibiotic application(multiple answer):  □On arrival  □During illness  □Continuous  □Periodical | | | |
| 21 | If you have not used antibiotics within last 14 days, did you use antibiotics in this chicken production cycle:  □Yes □No | | | |
| 22 | Use of antibiotics (**on selling day**): □Yes □No | | | |
| 23 | Use of growth promoter / probiotic containing antimicrobials:  □Yes □No | | | |
|  | If yes, mention the name of the antimicrobials (generic name) used in growth promoter / probiotic:  □__________________________□________________________  □__________________________□________________________ | | | |
|  | Purposes:□Prophylactic □Therapeutic □Both | | | |
|  | Timing of application: □On arrival □During illness □Continuous □Periodical | | | |
| 24 | Who suggest to use antimicrobials (multiple answer)?:  □ Veterinary doctor  □ Poultry consultant  □ Drug seller  □ Chick supplier  □ Veterinary medical representative  □ Quack  □ Self decision  □ Others _________ | | | |
| 25 | How often you follow **doctor’s suggestion**  to use antibiotic:  □ Always  □ Sometimes  □ Most of the times  □ No  **Feed dealer’s** suggestion  □ Always  □ Sometimes  □ Most of the times  □ No  **Drug seller’s** suggestion  □ Always  □ Sometimes  □ Most of the times  □ No | | Pharmaceutical **company representative’s** suggestion  □ Always  □ Sometimes  □ Most of the times  □ No  Pharmaceutical **company vet doctor’s** suggestion  □ Always  □ Sometimes  □ Most of the times  □ No  Quack**’s** suggestion  □ Always  □ Sometimes  □ Most of the times  □ No  **Self decision**  □ Always  □ Sometimes  □ Most of the times  □ No | |
| 26 | Farmer’s gender: □ Male □ Female | | | |
| 27 | Farmer’s highest educational degree:__________ | | | |
| 28 | Farmer’s experience in chicken Farming: ______________year | | | |
| 29 | Received formal training about poultry management: □ Yes □ No | | | |
| 30 | Do you know about the antimicrobial resistance (AMR): □ Yes □ No | | | |
| 31 | Do you know about the withdrawn period of antibiotics: □ Yes □ No | | | |
|  | If yes, how long_____________days | | | |
| 32 | Why antibiotics are used (multiple answer)?  □ To treat all diseases  □ To treat bacterial diseases  □ To treat viral disease  □ To increase production  □ Others________________ | | | |
| 33 | Presence of antibiotics (spot-check) at farms:  □_____________________□_____________________□____________________□___________ | | | |
| 34 | Presence of probiotic/growth promoter (spot-check) at farms:  □_____________________□_____________________□____________________□___________ | | | |
| 35 | Presence of other antimicrobial (anti-coccidial, anti-viral, anthelmintics) (spot-check) at farms:  □_____________________□_____________________□____________________□___________ | | | |
| 36 | Sources of antibiotics (multiple answer):  □ Animal feed dealers  □ Veterinary pharmacy  □ Sales representative (pharmaceutical company)  □ Veterinarian  □ Quack  □ Others______________ | | | |
| 37 | Average cost for antibiotics per batch-cycle  □ Broiler______________BDT/1000 chicken  □ Layer_______________BDT/1000 chicken  □ Sonali______________BDT/1000 chicken  □ Other______________BDT/1000 chicken | | | |
| 38 | Is your chicken production associated with other external factors? (multiple answers)  □ Credit (dependent on feed dealers)  □ Credit (dependent on big poultry industries)  □ Credit (dependent on poultry hatcheries)  □ Contract basis (dependent on feed dealers)  □ Contract basis (dependent on big poultry industries)  □ Contract basis (dependent on poultry hatcheries)  □ Independent (no dependency on feed dealers/industries/hatcheries)  □ Others_____________________________________ | | | |
