# Supplementary material for: Antibiotic usage practices and its drivers in commercial chicken production in Bangladesh
Source: PLoS One. 2022 Oct 17;17(10):e0276158. doi: 10.1371/journal.pone.0276158 (PMC9576089; doi:10.1371/journal.pone.0276158)
Supplement: S2 File — (DOCX) [file pone.0276158.s002.docx]

**Supplement 2:** Questionnaire for AMU data collection from animal feed dealer and farmers

| 1 | Pharmacy ID: | | | Date: |  |
| --- | --- | --- | --- | --- | --- |
| 2 | Pharmacy name: | | | Address: |  |
| 3 | Phone number: | | |  |  |
| 4 | GPS (feed dealer’s location) | | |  |  |
| 5 | Type of farmers: □ Layer farmer □ Broiler farmer □ Aquaculture farmer | | | |  |
| 6 | Purposes of feed dealer visit by farmers (multiple answers):  □ To collect medicine  □ To collect day-old chicks  □ To collect fingerling fish  □ To treat chicken  □ To get suggestions on treatment  □ To get suggestions on production increase  □ To get suggestions on management  □ To sale chicken product  □ To sale fish product  □ Others______________ | | | |  |
| 7 | If drug dispensed to the farmers, name of drugs (multiple answer)  □ Antibiotic  □Antiprotozoa  □Antiparasitic  □Vitamin  □Mineral  □Growth promoter  □Probiotic  □Others_________________ | | | |  |
| 8 | Drug dispensed for:  □ Broiler  □ Layer  □ Sonali  □ Aquaculture  □Others__________________ | | | |  |
| 9 | Antibiotics dispensed: □Yes □No | If yes, mention the name of the antibiotics (generic name):  □__________________________□_____________________  □__________________________□_____________________ | | |  |
|  |  | Purposes of antibiotic use:  □ Prophylactic  □ Therapeutic  □ Both | | |  |
|  |  | Who suggest to use antimicrobials (multiple answer)?:  □ Veterinary doctor  □ Poultry consultant  □ Fishery officer  □ Drug seller  □ Chick supplier  □ Fingerling supplier  □ Veterinary medical representative  □ Quack  □ Self decision  □ Others _________________ | | |  |
| 10 | How often you follow **doctor’s suggestion** to use antibiotic:  □ Always  □ Sometimes  □ Most of the times  □ No  How often you follow **fishery officer’s suggestion** to use antibiotic:  □ Always  □ Sometimes  □ Most of the times  □ No  **Feed dealer’s** suggestion  □ Always  □ Sometimes  □ Most of the times  □ No  **Drug seller’s** suggestion  □ Always  □ Sometimes  □ Most of the times  □ No | | Pharmaceutical **company representative’s** suggestion  □ Always  □ Sometimes  □ Most of the times  □ No  Pharmaceutical company **vet doctor’s** suggestion  □ Always  □ Sometimes  □ Most of the times  □ No  Pharmaceutical company **quack’s** suggestion  □ Always  □ Sometimes  □ Most of the times  □ No  **Self decision**  □ Always  □ Sometimes  □ Most of the times  □ No | |  |
| 11 | Farmer’s gender: □ Male □ Female | | | |  |
| 12 | Farmer’s highest educational degree:________________________ | | | |  |
| 13 | Farmer’s experience in chicken/aquaculture farming: ______________year | | | |  |
| 14 | Received formal training about chicken/aquaculture management: □ Yes □ No | | | |  |
| 15 | Does farmer know about the antimicrobial resistance (AMR): □ Yes □ No | | | |  |
| 16 | Does farmer know about the withdrawn period of antibiotics: □ Yes □ No | | | |  |
|  | If yes, how long__________days | | | |  |
| 17 | Does feed dealer know about the antimicrobial resistance (AMR): □ Yes □ No | | | |  |
| 18 | Does feed dealer know about the withdrawn period of antibiotics: □Yes □No | | | |  |
|  | If yes, how long____________days | | | |  |
| 19 | List of antimicrobials (generic name) at feed dealer shop(spot-check):  □__________________________□________________________□_______________________  □__________________________□________________________□_______________________ | | | |  |
| 20 | Any negotiation between farmer and feed dealer:  □ Yes □ No  If yes, What types of support (multiple answer)  □ Supply day-old chick  □ Supply fingerling fish  □ Supply feed  □ Supply medicine  □ Credit support  □ Others_____________________ | | | | |
| 21 | Where farmer’s sale chicken products (multiple answer)?  □ Local market  □ Wholesale market  □ Feed dealer  □ Middlemen  □ Others______________ | | | | |
| 22 | Where you sale fishes (multiple answer)?  □ Local market  □ Wholesale market  □ Feed dealer  □ Middlemen  □ Others______________ | | | | |
| 23 | Is your (farmer) chicken/fish production associated with other external factors? (multiple answers)  □ Credit (dependent on feed dealers)  □ Credit (dependent on big poultry/fish industries)  □ Credit (dependent on poultry/fish hatcheries)  □ Contract basis (dependent on big poultry/fish industries)  □ Contract basis (dependent on poultry/fish hatcheries)  □ Independent (no dependency on feed dealers/industries/hatcheries)  □ Others_____________________________________ | | | | |
