# Supplementary material for: Antibiotic usage practices and its drivers in commercial chicken production in Bangladesh
Source: PLoS One. 2022 Oct 17;17(10):e0276158. doi: 10.1371/journal.pone.0276158 (PMC9576089; doi:10.1371/journal.pone.0276158)
Supplement: Attachment — Submitted filename: Manuscript_Reviewed April 2022.docx [file pone.0276158.s003.docx]

**Antibiotic Usage Practices and Its Drivers in Commercial Chicken Production in 3 districts of Bangladesh**

Sukanta Chowdhury^1^, Guillaume Fournié^2^, Damer Blake^2^, Joerg Henning^3^, Patricia Conway^4^, Md. Ahasanul Hoque^5^, Sumon Ghosh^1^, Shahana Parveen^1^, Paritosh Kumar Biswas^5^, Zubair Akhtar^1^, Khaleda Islam^6^, Md. Ariful Islam^1^, Md. Mahbubur Rashid^1^, Ludvic Pelligand^2^, Zobaidul Haque Khan^6^, Mahmudur Rahman^7^, Fiona Tomley^2^, Nitish Debnath^6^ and Fahmida Chowdhury^1^

^1^International Centre for Diarrhoeal Disease Research, Bangladesh (icddr,b)

^2^Royal Veterinary College, UK

^3^The University of Queensland, Australia

^4^Nanyang Technological University, Singapore

^5^Chattogram Veterinary and Animal Sciences University

^6^Fleming Fund Country Grant to Bangladesh, DAI Global, LLC, Dhaka, Bangladesh

^7^Global Health Development, EMPHNET, Dhaka, Bangladesh

**Abstract**

Irrational and inappropriate use of antibiotics in commercial chicken production can contribute to the development of antimicrobial resistance. We aimed to assess antimicrobial usage practices in commercial chicken production in Bangladesh, and identify factors associated with this practice. We conducted a large-scale cross-sectional study to collect information on antibiotic usage in commercial chickens from January to May 2021. Structured interviews were conducted with 288 broiler, 288 layer and 192 Sonali (locally-produced cross-bred) farmers in 20 sub-districts across Bangladesh (convenience sample). The frequency of antibiotic usage, the types of antibiotics and purpose of usage were estimated for each production type. Adjusted odds ratios (aOR) were calculated to measure the association between antibiotic usage and factors related to the characteristics of the farms and farmers using multivariable logistic regression models. The proportion of farms, irrespective of their production type, reporting usage of antibiotics in the 24 hours preceding the interview was 41% (n=314, 95% CI: 37-44%). Forty-five percent (n=344, 41-48%) reported antibiotic usage in the last 72 hours, 86% (n=658, 83-88%) in the last 14 days, and almost all farms, 98% (n=753, 97-99%), had used antibiotics since the start of their production cycle. Use of antibiotics in the 24 hours preceding an interview was more frequently reported in broiler (OR 1.91, 95% CI: 1.36-2.69) and Sonali (OR 1.94, 95% CI: 1.33-2.33) than layer farms. Oxytetracycline (23-31%, depending on production type), doxycycline (18-25%), ciprofloxacin (16-26%) and amoxicillin (16-44%) were the most frequently used antibiotics. Antibiotics were reported to be used for both treatment and prophylactic purposes on most farms (57-67%). Usage of antibiotics in the 24h preceding an interview was significantly associated with the occurrence of any illnesses in chickens (aOR broiler: 41.22 [95% CI:13.63-124.62], layer: aOR 36.45[9.52-139.43], Sonali: aOR 28.47[4.97-162.97]). Antibiotic usage was mainly advised by veterinary practitioners (45-71%, depending on production type), followed by feed dealers (21-40%) and farmers (7-13%). Improvement of chicken health through good farming practices along with changes in key stakeholders (feed dealers and practitioners) attitudes towards antibiotic recommendations to farmers, may help to reduce the levels of antibiotic usage and thus contribute to mitigate antimicrobial resistance.

**Keywords:** Antibiotics, commercial chicken, Bangladesh

**Introduction**

Globally, antimicrobial resistance (AMR) is one of the greatest threats to public health. Inappropriate use of antimicrobials in humans, poultry, fish, and livestock has contributed to AMR emergence[1, 2]. Antimicrobials are frequently used as prophylactic drugs in commercial animal production systems in low and middle income countries[3]. While a study suggests that global consumption of antimicrobials in animals will rise by 67% by 2030[4], there is no actual estimate of global antimicrobial consumption in animal production so far. According to expert opinions, antimicrobial consumption in animals is almost double that of human consumption[5]. Despite the benefit of treating animal diseases using antimicrobial drugs, the development of AMR in both animal and human associated bacterial populations has raised global concerns[6].

In Bangladesh, commercial chicken production is greatly expanding to meet the rising demand for meat and eggs for human consumption. The commercial chicken industry includes broiler, layer and Sonali intensive farms. Broilers and layers are exotic chickens reared for meat and eggs, respectively. Sonali is a locally-produced cross-bred between Rhode Island Red male and Fayoumi female, reared for both meat and egg production[7, 8]. Although the extent of antibiotic usage in poultry is not well explored, a small-scale study in Bangladesh reported that 98% of commercial chicken farms used antibiotics[9]. In 2010, the Bangladesh government passed the “Bangladesh Fish Feed and Animal Feed Act 2010” banning the introduction of antibiotics into animal feed during manufacturing[10]. Yet, the easy access and availability of over-the-counter antibiotics at feed dealer shops and pharmacies can play an important role for the emergence of AMR in Bangladesh[11]. A large number of animal feed dealers and drug sellers advise farmers to use antimicrobials for chicken production, despite limited understanding about the impact of excessive and prophylactic use of antibiotics on AMR emergence[12]. To date, Bangladesh has no drug policy or guideline for appropriate use of antibiotic to treat animals, except for the above-mentioned ban on the addition of antimicrobials during feed manufacture. Increasing information on current practices related to antibiotic usage in commercial chicken production is crucial for the design of more effective interventions to minimize the animal and public health impact of AMR. To address this gap, we conducted a cross-sectional study in intensive commercial exotic chicken production areas in Bangladesh to collect information about the nature of antibiotic usage in commercial broiler, layer and Sonali chicken farms.

### Materials and methods

**Study design**

A cross-sectional study was carried out to collect antibiotic usage information in commercial chicken farms in Bangladesh from January to May 2021. Farms were stratified based on their (i) location (upazilas, i.e. sub-districts), (ii) production type (broiler, layer or Sonali), and (iii) scale (small: ≤1000 chickens, medium: 1001-2000, large: >2000 chickens).

Upazilas (sub-districts) with the highest number of commercial chicken farms were chosen for this study, as follows: Gazipur, Chattogram, and Cumilla districts were selected for broiler and layer farms; and Joypurhat and Bogura districts for Sonali farms (Figure 1)[8]. Through consultation with local livestock officers from the Bangladesh Department of Livestock Services (DLS) and feed dealers, the 4 upazilas with the highest number of farms in each district were then selected. In each of these selected upazilas, the field team enrolled 8 small, 8 medium and 8 large farms for each targeted production type (broiler, layer and/or Sonali depending on the upazila). In the absence of a reliable list of commercial farms, a snow ball sampling approach was used to identify farms in each upazila. Feed dealers operating in the targeted upazilas were asked about the farms they do business with. A first farm was then recruited, and the farmer asked about the address of the nearest farm, which was then recruited. This procedure was repeated until reaching the targeted sample size for each production type and farm size category. Chicken flocks younger than 14 days were not included in this study, as we asked about antibiotic usage on the 14 days preceding the farm visit. Overall, 288 broiler and 288 layer farms were enrolled in 12 upazilas in 3 districts, and 192 Sonali farms were enrolled in 8 upazilas in 2 districts. The sample size was calculated assuming a 32% expected prevalence of antibiotic prophylactic usage in broiler farms[13], with 95% level of significance and 5% precision.

A total of 20 animal feed dealers were interviewed from the 20 above-mentioned selected upazilas. In each upazila, a large feed dealer was selected. We purposively selected feed dealers selling larger amount of poultry feed and medicine. The field team visited each selected feed dealer once (preferably during the busiest hours, as farmers visited their shop) to record drug dispensing practices by observing interactions with five consecutive chicken farmers. A total of 100 (five farmers per feed dealer) interactions with farmers were thus observed. Antibiotics available for sale in each shop were also recorded.

**Data collection**

A structured questionnaire was used to collect data. Before data collection, written informed consent was obtained from all selected farmers, animal feed and chick dealers to participate in the study. We collected data on farm demographics, production stage, antibiotic usage in the 24h, 72h and 14 days preceding the interview, antibiotic usage since the start of the production cycle, name of antibiotics used, pro-biotic usage, purpose(s) of antibiotic usage, antibiotic prescribing practices by authorized practitioners, chicken morbidity during the day of the farm visit, chicken mortality over the last14 days, farmer’s education, duration of farming experience and familiarity with the term “AMR". The questionnaire for animal feed dealers covered antibiotic dispensing practices, available antibiotics kept for sale, antibiotic prescribing practices by authorized practitioners and familiarity with the term “AMR".

**Statistical analysis**

We summarized the characteristics of chicken farms, including flock size and production type, using descriptive analyses. Fisher’s exact test was performed to examine the differences of proportion of demographic characteristics between categories of each categorical variable. One-way ANOVA was conducted to assess the mean difference of health status, flock and farm size between categories of each categorical variable. The proportion of farms using each type of antibiotic and its 95% confidence interval was estimated separately for each production type. To describe the association between categorical farm management or demographic or geographic variables, and the use of antibiotics on chicken farms, we firstly estimated the odds ratio (OR) using bivariable logistic regression analysis. Then, we performed multivariable analyses to estimate adjusted odds ratio (aOR) for each production type using additional explanatory variables. Variables with a *p* value ≤0.2 at a likelihood ratio test were used to build a multivariable logistic regression model. We used backward stepwise selection of variables with an inclusion threshold of 0.05. We used Hosmer-Lemeshow test to calculate model χ^2^ statistic and McFadden’s pseudo-R^2^ (the coefficient of determination) to explain variance and measure goodness-of-fit for multivariate regression model. All statistical analyses were performed in Stata 13 software (StataCorp LP, College Station, TX).

**Results**

**Demographic characteristics of commercial chicken farms**

Among the 768 surveyed commercial chicken farms, most farms (88-91% depending on production type) only raised one production type, and 9-12% of farms raised different types of chickens. About half of farms (44-61%) had a single poultry shed. Most farms (69-92%) had a single batch of chickens during our visit. Almost all farms (93-100%) collected feed and most (51-75%) collected day-old chicks from feed and/or chick dealers. Others prepared feed at their farms and collected day-old chicks from hatcheries. During the farm visits, the average age of broiler, layer and Sonali chickens was 22 days , 322 days and 91 days , respectively (Table 1).

**Frequency and characteristics of antibiotic usage within the last 24 hours**

Among the 768 commercial chicken farms, 41% (n=314, 95% CI: 37-44%) reported having used antibiotics within the 24 hours preceding our visit. This proportion was higher among broiler (47%) and Sonali (47%) than in layer farms (31%) (Table 2). Two-third of farms (66-67% depending on production type) administered antibiotics for both therapeutic and prophylactic purposes. The proportion of farms that used antibiotic prophylactically and not therapeutically was slightly higher in layer (14%) than broiler (8%) and Sonali (3%) farms. Commercial chicken farms used diverse classes of antibiotics including tetracyclines, fluoroquinolones, macrolides, aminoglycosides, penicillins, and polymyxins. Doxycycline, oxytetracycline and ciprofloxacin were the most commonly reported antibiotics in broiler and layer chickens, whereas amoxicillin usage was more commonly reported in Sonali chickens (Table 3). According to reports from farmers, antibiotics were mostly prescribed by veterinary practitioners (55%), followed by poultry feed dealers (30%) and the farmers themselves (9%). Broiler farmers relied more frequently on feed dealer’s advice about antibiotic usage than layer and Sonali chicken farmers (Table 2).

**Frequency and characteristics of antibiotic usage earlier in the production cycle**

Almost all farms (98%, 95% CI: 97-99%, n=753) had used antibiotics since the start of the production cycle. In the 72 hours and 14 days preceding the interview, 45% (n=344, 95% CI 41-48%) and 86% (n=658, 95% CI 83-88%) of farms reported having used antibiotics, respectively (Tables 4 and 5). The purposes of antibiotic usage in the last 14 days were similar to those reported for the last 24 hours, with most farmers (57-67% depending on production type) administering antibiotics for both prophylactic and therapeutic usage. The frequency of prophylactic usage of antibiotics only was comparatively higher in layer (16%) than broiler (8%) and Sonali (3%) farms. Likewise, similarly to antibiotics used in the last 24 hours, antibiotics used in the last 14 days were mostly prescribed by veterinary practitioners (59%), followed by poultry feed dealers (29%) and farmers themselves (7%). Broiler farmers relied more frequently on feed dealer’s advice about antibiotic usage than layer and Sonali chicken farmers (Table 5).

**General practices with regards to antibiotic usage**

More than 90% of commercial chicken farmers used antibiotics in their flocks for 3-7 consecutive days. Most farmers used antibiotics on the first day of the batch production cycle therapeutically (83-86%), or prophylactically (21-55%). Many farmers (16-41%) reported using antibiotics for chicken fattening (growth promotion). While the majority (67-87%) of the farmers had heard about withdrawal periods for antibiotics, 3-5% of farmers planned to use antibiotics on the last day of the production cycle, just before sales. A few layer (n=11, 4%) and Sonali farmers (n= 12, 6%) reported that they mixed antibiotics into the chicken feed for administration. There was no history of mixing antibiotic in chicken feed by broiler farmers. According to farmers’ estimations, the mean cost of antibiotics per production cycle was 80 USD (standard deviation, SD ±58), 541 USD (SD ±472) and 172 USD (SD ± 151) for 1000 broiler, layer and Sonali chicken, respectively.

**Health status of commercial chicken flocks**

On the day of farm visit, 103 broiler (36%), 58 layer (20%) and 69 Sonali (36%) farms had at least one sick chicken. Most surveyed farms reported that at least one chicken was sick (97% broiler,74% layer and 85% Sonali farms) and/or died (97% broiler, 67% layer and 85% Sonali farms) within the 14 days preceding our farm visit. The overall proportion of sick chickens per farm on the day of farm visit? was 71 per 1000 broiler, 21 per 1000 layer and 31 per 1000 Sonali farms, whereas the overall proportion of dead chickens was 39 per 1000 broiler, 9 per 1000 layer and 23 per 1000 Sonali chickens.

**Interaction between farmers, feed dealers and other associated partners**

Most farmers (58-80%) had interactions with feed dealers. Farmers received support from feed dealers mainly on feed supply, followed by chick supply, medicine supply, health care services with or without vets, sale of mature chickens and eggs, and the provision of credit. According to the farmers’ reports, chicken production depended on credit from feed dealers (22-36% farms) and pre-existing agreements (other than credit including chick supply, feed supply and sale mature chicken) with feed dealers (25-41% farms). Layer farms were less dependent on such arrangement than broiler and Sonali farms (Table 6).

**Antibiotic dispensing at feed dealers**

During visits to the shops of feed dealers, vitamins (72%, n=72) were most commonly dispensed to farmers, followed by antibiotics (41%, n=41) and probiotics (30%, n=30). Farmers bought medicines for broiler (42%), Sonali (40%) and layer (18%) chickens. Among the farmers purchasing antibiotics, most were advised by qualified veterinarians (n=22, 54%), feed dealers (n=2, 5%), or the decision was based on their own experience (n=17, 41%). Six to twelve classes of antibiotics were available in visited feed dealers’ shop. According to the self-reported data, 70% of feed dealers usually suggest 10-30% of farmers to buy antibiotics. Most feed dealers (85%) said they knew about AMR and all had knowledge on antibiotic withdrawal periods. Many feed dealers (60%) believed that antibiotics are mixed in poultry feed by commercial feed producers despite understanding that antibiotic use in commercial poultry feed is banned by the Bangladesh government.

**Factors associated with antibiotic usage in commercial chicken flocks in the 24 hours preceding the farm visit**

The overall usage of antibiotics in the previous 24 hours appeared to be higher in broiler (OR 1.91, 95% CI: 1.36-2.69) and Sonali (OR 1.94, 1.33-2.83) than layer farms. The odds of antibiotic usage were higher in medium (OR 1.41, 0.99-2.01) and large (OR 1.39, 0.97-1.98) than small farms (Table 7). The bivariable regression analysis showed that broiler (OR 2.79, 0.93-8.34) and layer (OR 3.09, 1.05-9.08) chicken farms located in Gazipur were more likely to use antibiotics than those in Cumilla (Tables 8 and 9). Sonali chicken farms located in Bogura (OR 3.67, 1.15-11.7) were more like to use antibiotics than those in Joypurhat (Table 10).

The multivariable regression analyses suggested that the occurrence of illness of any type (at least one sick chicken within the preceding 24 hours) was associated with higher odds of antibiotic usage in broiler (aOR 41.22, 13.63-124.62), layer (aOR36.45, 9.52-139.43) and Sonali farms (aOR28.47, 4.97-162.97). The odds were also higher for broiler (aOR 2.33, 1.02-5.33) and layer (aOR3.79, 1.71-8.38) farms located in Gazipur than Cumilla district, as well as for broiler farms for which the farmers had inappropriate knowledge on antibiotic use (aOR 3.25, 1.69-6.26) (Tables 8-9). The final model selected for layer (χ2 10.67, *p*=0.64, and R2=0.33) and Sonali (χ2 6.86, *p*=0.14, and R^2^0.38) seemed to fit the data well, whereas the model selected for broiler (χ2 20.81, *p*=0.06, and R2=0.39) did not fit as well (Tables 8, 9 and 10).

**Discussion**

This study surveyed a large number of commercial chicken farms from a wide range of geographical locations in Bangladesh, including different chicken production types and scales (small to large). This study showed that commercial chicken farmers frequently administer antibiotics to chickens, in particular broiler and Sonali chickens. Under this study, antibiotic usage data was collected over different timeframes, in the 24 hours, 72 hours and 14 days preceding our farm visit. No previous published studies have reported antibiotic usage in commercial chicken production using similar time frames for Bangladesh. Few previous studies from Bangladesh have estimated the proportion of farms using antibiotics, but accurate comparison between production systems has been precluded in the absence of detailed timeframes. It has been reported that 54%-100% of broiler and layer farms administer antibiotics from the start of the production cycle to the day they were surveyed [9, 14, 15]. Antibiotic usage in commercial poultry in many low- and middle-income countries including Sudan, Tanzania, Vietnam, Philippines, Pakistan, Nepal, Ghana, Nigeria and Cameroon varied from 44-100%, either at the time of farm visits or during the chicken production cycle [16-24]. For comparison, the proportion of broiler chicken farms using antimicrobials was 26% on day 1 and 49% within the first week of production in nine European countries [25].

According to the World Organization for Animal Health (OIE) list of antimicrobial agents of veterinary importance, many of the antibiotics reported to have been used in recruited farms (including doxycycline, oxytetracycline, amoxicillin, neomycin, erythromycin, tylosin, ciprofloxacin, enrofloxacin) are considered as Veterinary Critically Important Antimicrobial Agents (VCIA). Among these VCIA, ciprofloxacin and enrofloxacin are also considered to be critically important for human health[26]. Previous studies conducted in Bangladesh, Pakistan, Vietnam, Philippines, Tanzania, Pakistan, Ghana, Nigeria and Cameroon have also reported common usage of such antibiotics of critical importance for animal and/or human health in commercial broiler and layer chickens[9, 27][20][17-19, 22, 28, 29].The usage of antibiotics in animal production systems is a global issue. Some antibiotics (colistin, fluoroquinolones and third- and fourth-generation cephalosporins) are advised not to be used in food-producing animals[30]. The World Health Organization (WHO) has recommended complete restriction of all classes of medically important antibiotics in food producing animals for prophylactic purposes[30]. Surprisingly, many countries including Bangladesh (17-57%), Pakistan (60%), Thailand (63%) and Vietnam (36%), as well as across nine European countries (18-26%), have reported use of critically important antimicrobial classes in commercial poultry production [9, 21, 25, 31, 32]. Interestingly, our study also identified a large number broiler, layer and Sonali farms that had used highest priority critically important antimicrobial classes such as colistin and fluoroquinolones. This extensive use of medically important antibiotics in commercial chicken production may promote the development of resistance in microbial populations infecting animals and humans.

Recognizing that use of antibiotics for prophylactic and growth promotion purposes in chicken production sectors is a matter of concern worldwide, our study recorded the frequent prophylactic usage of antibiotics in commercial farms. Many farmers reported that they used antibiotics for fattening purposes (growth promotion). Earlier studies from Bangladesh reported similar evidence of antibiotic use for prophylaxis (23-32%) and growth promotion (8%) in commercial chicken production [13, 15]. The routine use of antibiotics at different stages of the production cycle in commercial chicken for prophylactic purposes has also been reported in Cameroon (11%), Pakistan (100%), Nigeria (29-60%), Nepal (22%) and Thailand (38%) [19, 20, 23, 24, 29, 32]. OIE and WHO advise to avoid antimicrobials for prophylactic purposes in the absence of clinical signs in food-producing animals[26, 30]. In parallel, the Bangladesh government passed a law in 2010 to ban the introduction of antibiotics into animal feed during manufacturing[10]. However, no guidelines or policies are available regarding the appropriate use of antibiotics in animal production sectors. The regular usage of antibiotics for prophylactic and growth promotion purposes in healthy animals can play a significant role in the emergence of antibiotic resistance[33].

This study identified some factors that were associated with increased antibiotic usage in commercial chicken production systems. Concurrent chicken morbidity, farm location, inadequate knowledge about the effect of antibiotics and poor understanding about AMR were significantly associated with increased antibiotic usage in commercial chickens. A study from China reported that lower education levels of farmers and lack of formal agricultural training, likely resulting in poor understanding of AMR, were associated with misuse of antibiotics in chicken farms [34]. According to farmers interviewed in this study, chicken illness was frequently occurring during our farm visit. To treat sick chickens, farmers were often advised to use antibiotics. Farms located in Gazipur and Bogura used comparatively more antibiotics than in other areas. However, reasons for this were not clearly understood. There might be higher prevalence of diseases in these areas due to high density of chicken farms with different level of biosecurity. Therefore, farmers in these areas could use more antibiotics than other areas.

This study revealed that a large proportion of farmers followed the advice of feed dealers about antibiotic usage. A large number of farmers were financially covenanted to feed dealers that may develop dependency of farmers to feed dealers. As feed dealers also sell antibiotics, they may encourage their purchase by farmers for business interest. Although most of the feed dealers were familiar with AMR, they recommended farmers buying antibiotics for their chickens. Antibiotic prescription by unqualified antibiotic providers needs to be controlled to minimize inappropriate use of antibiotics in commercial chicken production sectors.

This cross-sectional survey may have some limitations. We used purposive sampling instead of random sampling to select farms in each sampling stratum. Limited time and funding did not allow us to conduct a census of all farms in each selected upazila to support their random sampling. However, this may not have influenced the study findings as the farm characteristics and reported antimicrobial usage were consistent with an earlier study[9]. The information that we collected from farmers about antibiotic usage may have been affected by social desirability; this may have resulted in an underestimation of the usage of antibiotics.

In conclusion, this cross-sectional survey revealed that the use of antibiotics in commercial chicken production was extensive in Bangladesh. Most antibiotics were administered for therapeutic and prophylactic purposes. Antibiotics were more commonly used in broiler and Sonali than in layer farms. The occurrence of antibiotic use in the 24 hours preceding our visit was significantly higher in flocks with clinically sick chickens than in healthy flocks. The findings from this study emphasize that the improvement of chicken health through good farming practices can help to reduce antibiotic use and the consequential development of antimicrobial resistance. Continuous monitoring of antibiotic usage, educating farmers, drug sellers and feed dealers about effective use of antibiotics, and restricting ease of access to antibiotics, may also be useful to reduce unnecessary use of antibiotics in commercial chicken production systems.

**Acknowledgements**

We are grateful to chicken farmers and feed dealers for providing their valuable time and information. The icddr,b is also grateful to the governments of Bangladesh, Canada, Sweden, and the United Kingdom for providing core/unrestricted support.

**Conflict of interest**

The authors have no conflict of interest to declare. The funders had no conflict of interest with the study and contents of the manuscript.

**Funding**

This study was funded by the Fleming Fund Country Grant to Bangladesh (FF48-416FFCGB1).

**Ethical Approval**

The study protocol was reviewed and approved by icddr,b Research Review Committee, Ethical Review Committee and Animal Experimentation Ethics Committee (PR-20116).

**Data Availability Statement**

The data of this study are available from the corresponding author upon reasonable request.

**References**

1.	Jansen, W., et al., *Bacterial resistance: a sensitive issue: complexity of the challenge and containment strategy in Europe.* Drug Resistance Updates, 2006. **9**(3): p. 123-133.

2.	Smolinski, M., M. Hamburg, and J. Lederberg, *Microbial threats to health: emergence.* Detection, and Response (Natl. Acad. Press, Washington, DC), 2003.

3.	Ali, H., et al., *An assessment of chemical and biological product use in aquaculture in Bangladesh.* Aquaculture, 2016. **454**: p. 199-209.

4.	Van Boeckel, T.P., et al., *Global trends in antimicrobial use in food animals.* Proceedings of the National Academy of Sciences, 2015. **112**(18): p. 5649-5654.

5.	Aarestrup, F., *Get pigs off antibiotics.* Nature, 2012. **486**(7404): p. 465-466.

6.	Landers, T.F., et al., *A review of antibiotic use in food animals: perspective, policy, and potential.* Public health reports, 2012. **127**(1): p. 4-22.

7.	Hennessey, M., et al., *Intensification of fragility: poultry production and distribution in Bangladesh and its implications for disease risk.* Preventive Veterinary Medicine, 2021. **191**: p. 105367.

8.	Dolberg, F., *Poultry sector country review, Bangladesh.* [*http://www.fao.org/3/ai319e/ai319e.pdf*](http://www.fao.org/3/ai319e/ai319e.pdf) *[Accessed on 25 February 2021].*

9.	Imam, T., et al., *A cross-sectional study of antimicrobial usage on commercial broiler and layer chicken farms in Bangladesh.* Frontiers in veterinary science, 2020. **7**.

10.	*Bangladesh Gazette. Fish Feed and Animal Feed Act 2010.* [*http://extwprlegs1.fao.org/docs/pdf/bgd165024.pdf*](http://extwprlegs1.fao.org/docs/pdf/bgd165024.pdf) *[Accessed April 20, 2021].*

11.	Rousham, E.K., et al., *Pathways of antibiotic use in Bangladesh: qualitative protocol for the PAUSE study.* BMJ open, 2019. **9**(1): p. e028215.

12.	Kalam, M., et al., *Knowledge, Attitude, and Practices on Antimicrobial Use and Antimicrobial Resistance among Poultry Drug and Feed Sellers in Bangladesh.* Veterinary Sciences, 2021. **8**(6): p. 111.

13.	Islam, K.S., S. Shiraj-Um-Mahmuda, and M. Hazzaz-Bin-Kabir, *Antibiotic usage patterns in selected broiler farms of Bangladesh and their public health implications.* Journal of Public Health in Developing Countries, 2016. **2**(3): p. 276-284.

14.	Ferdous, J., et al., *Assessing farmers’ perspective on antibiotic usage and management practices in small-scale layer farms of Mymensingh district, Bangladesh.* Veterinary world, 2019. **12**(9): p. 1441.

15.	Tasmim, S., et al., *Socio-demographic determinants of use and misuse of antibiotics in commercial poultry farms in Bangladesh.* International Journal of Infectious Diseases, 2020. **101**: p. 90.

16.	Sirdar, M.M., et al., *A questionnaire survey of poultry layer farmers in Khartoum State, Sudan, to study their antimicrobial awareness and usage patterns.* Onderstepoort Journal of Veterinary Research, 2012. **79**(1): p. 1-8.

17.	Nonga, H.E., et al., *Assessment of antimicrobial usage and antimicrobial residues in broiler chickens in Morogoro Municipality, Tanzania.* Pakistan journal of Nutrition, 2009. **8**(3): p. 203-207.

18.	Barroga, T.R.M., et al., *Antimicrobials used in backyard and commercial poultry and swine farms in the Philippines: a qualitative pilot study.* Frontiers in veterinary science, 2020. **7**: p. 329.

19.	Kamini, M.G., et al., *Antimicrobial usage in the chicken farming in Yaoundé, Cameroon: a cross-sectional study.* International Journal of Food Contamination, 2016. **3**(1): p. 1-6.

20.	Umair, M., et al., *Quantification and Trends of Antimicrobial Use in Commercial Broiler Chicken Production in Pakistan.* Antibiotics, 2021. **10**(5): p. 598.

21.	Cuong, N.V., et al., *High-resolution monitoring of antimicrobial consumption in Vietnamese small-scale chicken farms highlights discrepancies between study metrics.* Frontiers in veterinary science, 2019. **6**: p. 174.

22.	Boamah, V.E., et al., *Practices and factors influencing the use of antibiotics in selected poultry farms in Ghana.* 2016.

23.	Oluwasile, B., et al., *Antibiotic usage pattern in selected poultry farms in Ogun state.* Sokoto Journal of Veterinary Sciences, 2014. **12**(1): p. 45-50.

24.	Koirala, A., et al., *Antibiotic Use in Broiler Poultry Farms in Kathmandu Valley of Nepal: Which Antibiotics and Why?* Tropical medicine and infectious disease, 2021. **6**(2): p. 47.

25.	Joosten, P., et al., *Quantitative and qualitative analysis of antimicrobial usage at farm and flock level on 181 broiler farms in nine European countries.* Journal of Antimicrobial Chemotherapy, 2019. **74**(3): p. 798-806.

26.	OIE, *OIE Standards, Guidelines and Resolutionon antimicrobial resistanceand the use of antimicrobial agents.* [*https://web.oie.int/delegateweb/eng/ebook/AF-book-AMR-ANG_FULL.pdf?WAHISPHPSESSID=03152ead00d06990fa9066b7b71fcabc*](https://web.oie.int/delegateweb/eng/ebook/AF-book-AMR-ANG_FULL.pdf?WAHISPHPSESSID=03152ead00d06990fa9066b7b71fcabc) *[accessed June 30, 2021].*

27.	Rousham, E.K., et al., *Human Colonization with Extended-Spectrum Beta-Lactamase-Producing E. coli in Relation to Animal and Environmental Exposures in Bangladesh: An Observational One Health Study.* Environmental health perspectives, 2021. **129**(3): p. 037001.

28.	Choisy, M., et al., *Assessing antimicrobial misuse in small-scale chicken farms in Vietnam from an observational study.* BMC veterinary research, 2019. **15**(1): p. 1-10.

29.	Adebowale, O.O., et al., *Antibiotic use and practices in commercial poultry laying hens in Ogun State Nigeria.* Revue d’élevage et de médecine vétérinaire des pays tropicaux, 2016. **69**(1): p. 41-45.

30.	Aidara-Kane, A., et al., *World Health Organization (WHO) guidelines on use of medically important antimicrobials in food-producing animals.* Antimicrobial Resistance & Infection Control, 2018. **7**(1): p. 1-8.

31.	Persoons, D., et al., *Antimicrobial use in Belgian broiler production.* Preventive veterinary medicine, 2012. **105**(4): p. 320-325.

32.	Wongsuvan, G., et al., *Antibiotic use in poultry: a survey of eight farms in Thailand.* Bulletin of the World Health Organization, 2018. **96**(2): p. 94.

33.	Thakur, S.D. and A. Panda, *Rational use of antimicrobials in animal production: a prerequisite to stem the tide of antimicrobial resistance.* Current Science, 2017: p. 1846-1857.

34.	Xu, J., et al., *Antibiotic use in chicken farms in northwestern China.* Antimicrobial Resistance & Infection Control, 2020. **9**(1): p. 1-9.

**Table 1:** *Demographic and health characteristics of commercial broiler (n=288), layer (n=288) and Sonali (n=192) chicken farms sampled during January to May 2021 in Bangladesh. Statistically significant differences are indicated within each characteristic.*

| **Characteristics** | **Broiler farms** | | | | **Layer farms** | | | | **Sonali farms** | | | |
| --- | --- | --- | --- | --- | --- | --- | --- | --- | --- | --- | --- | --- |
| **Areas, n (%)** | Small | Medium | Large | *Overall p* | Small | Medium | Large | *Overall p* | Small | Medium | Large | *Overall p* |
| Gazipur | 32 (11) | 32 (11) | 32 (11) |  | 32 (11) | 32 (11) | 32 (11) |  | - | - | - |  |
| Chattogram | 32 (11) | 32 (11) | 32 (11) |  | 32 (11) | 32 (11) | 32 (11) |  | - | - | - |  |
| Cumilla | 32 (11) | 32 (11) | 32 (11) |  | 32 (11) | 32 (11) | 32 (11) |  | - | - | - |  |
| Joypurhat | - | - | - |  | - | - | - |  | 32 (17) | 32 (17) | 32 (17) |  |
| Bogura | - | - | - |  | - | - | - |  | 32 (17) | 32 (17) | 32 (17) |  |
| **Number of batches of chickens, n (%)** | |  |  |  |  |  |  |  |  |  |  |  |
| Single | 96 (33) | 91 (32) | 79 (27) | <0.001 | 87 (30) | 76 (26) | 35 (12) | <0.001 | 63 (33) | 57 (30) | 32 (17) | <0.001 |
| Two | - | 5 (2) | 15 (5) |  | 9 (3) | 19 (7) | 46 (16) |  | 1 (1) | 7 (4) | 18 (9) |  |
| Three or more | - | - | 2 (1) |  | - | 1 (1) | 15 (5) |  | - | - | 14 (7) |  |
| **Number of poultry sheds, n (%)** | |  |  |  |  |  |  |  |  |  |  |  |
| Single | 74 (24) | 60 (21) | 42 (15) |  | 60 (21) | 42 (15) | 25 (7) |  | 48 (25) | 33 (17) | 6 (3) |  |
| Two | 20 (7) | 30 (10) | 35 (12) | <0.001 | 27 (9) | 34 (12) | 23 (8) | <0.001 | 12 (4) | 28 (10) | 22 (8) | <0.001 |
| More than two | 2 (1) | 6 (2) | 19 (7) |  | 9 (3) | 20 (7) | 48 (17) |  | 4 (1) | 3 (1) | 36 (13) |  |
| **Source of chicken feed, n (%)** | |  |  |  |  |  |  |  |  |  |  |  |
| Feed dealers | 96 (33) | 96 (33) | 96 (33) |  | 94 (33) | 91 (32) | 87 (30) |  | 62 (32) | 62 (32) | 55 (29) |  |
| Home made | - | - | - | - | 2 (1) | 5 (2) | 8 (3) | 0.129 | 2 (1) | 2 (1) | 9 (5) | 0.03 |
| Both sources | - | - | - |  | - | - | 1 (1) |  | - | - | - |  |
| **Collection of day-old chicks, n (%)** | | |  |  |  |  |  |  |  |  |  |  |
| Hatchery | 21 (7) | 19 (7) | 31 (11) |  | 41 (14) | 46 (16) | 54 (19) |  | 23 (12) | 18 (9) | 30 (16) |  |
| Feed and/or chick dealer | 75 (26) | 77 (27) | 65 (23) | 0.108 | 55 (19) | 50 (17) | 42 (15) | 0.177 | 41 (21) | 46 (24) | 34 (18) | 0.093 |
| **Health status of chicken flock, mean (standard deviation)** | | | | |  |  |  |  |  |  |  |  |
| Average number of sick chickens within last 14 days | 65  (117) | 88  (124) | 178  (226) | <0.001 | 68 (24) | 66 (22) | 77 (27) | 0.242 | 48 (25) | 55 (29) | 60 (31) | 0.245 |
| Average number of dead chickens within last 14 days | 36  (62) | 46  (65) | 104  (120) | <0.001 | 61 (21) | 59 (20) | 73 (25) | 0.057 | 48 (25) | 55 (29) | 60 (31) | 0.486 |
| **Average number of chickens/farm, mean (standard deviation)** | 782 (215) | 1451 (293) | 2975 (1289) | <0.001 | 831 | 1695 | 5432 | <0.001 | 893 | 1787 | 6152 | <0.001 |
| **Average size of farm shed in Sq-feet, mean (standard deviation)** | 1102 (601) | 1618 (689) | 3170 (6035) | <0.001 | 1305 | 2007 | 3349 | 0.002 | 1415 | 2100 | 2821 | 0.018 |
| **Average age of the chicken in days, mean (standard deviation)** | 22 (6) | 23 (9) | 22 (5) | 0.557 | 335 | 295 | 335 | 0.698 | 87 | 63 | 123 | 0.710 |

**Table 2:** *Farm-level antibiotic use within the 24 hours preceding visits to commercial broiler, layer and Sonali chicken farms in Bangladesh during January-May 2021.*

|  | **Broiler(N=288)** | | **Layer (N=288)** | | **Sonali (N=192)** | |
| --- | --- | --- | --- | --- | --- | --- |
| **Variables** | **Number of farms**  **n (%)** | **95% CI** | **Number of farms**  **n (%)** | **95% CI** | **Number of farms**  **n (%)** | **95% CI** |
| **Uses of at least one antibiotic** | 134 (47) | 41-52 | 90 (31) | 26-37 | 90 (47) | 40-54 |
| **Number of antibiotics** | **N=134** |  | **N=90** |  | **N=90** |  |
| Single antibiotic | 74 (55) | 46-63 | 44 (49) | 38-59 | 49 (54) | 44-65 |
| Two antibiotics | 48 (36) | 27-44 | 34 (38) | 27-48 | 36 (40) | 30-51 |
| Three or more antibiotics | 12 (9) | 4-15 | 12 (13) | 7-22 | 5 (6) | 2-12 |
| **Purposes of antibiotic use** | **N=134** |  | **N=90** |  | **N=90** |  |
| Prophylaxis | 11 (8) | 4-14 | 13 (14) | 8-24 | 3 (3) | 1-9 |
| Treatment | 34 (25) | 18-33 | 18 (20) | 14-32 | 27 (30) | 21-41 |
| Both | 89 (66) | 57-74 | 59 (66) | 54-75 | 60 (67) | 56-76 |
| **Antibiotic suggested by** | **N=134** |  | **N=90** |  | **N=90** |  |
| Veterinary practitioner | 60 (45) | 36-53 | 49 (54) | 43-64 | 64 (71) | 61-80 |
| Pharmacy owner | 1 (1) | 1-4 | 3 (3) | 1-9 | - | - |
| Feed dealer | 54 (40) | 31-49 | 20 (22) | 14-32 | 19 (21) | 13-31 |
| Veterinary doctor from pharmaceutical company | 1 (1) | 1-4 | 1 (1) | 1-6 | 1 (1) | 1-6 |
| Quack | 1 (1) | 1-4 | 11 (12) | 6-20 | - | - |
| Self-decision | 17 (13) | 7-19 | 6 (7) | 2-13 | 6 (7) | 2-14 |

**Table 3:** *Types of antibiotics used within the 24 hours preceding visits to broiler (N=134), layer (N=90) and Sonali(N=90) farms in Bangladesh during January-May 2021.*

| **Name of the antibiotic** | **Number of broiler farms**  **n (%)** | **Number of layer farms**  **n (%)** | **Number of sonali farms**  **n (%)** |
| --- | --- | --- | --- |
| **Tetracycline** |  |  |  |
| Doxycycline | 34 (25) | 27 (30) | 16 (18) |
| Oxytetracycline | 31 (23) | 28 (31) | 23 (26) |
| **Fluoroquinolones** |  |  |  |
| Ciprofloxacin | 30 (22) | 23 (26) | 14 (16) |
| Levofloxacin | 11 (8) | 7 (8) | 15 (17) |
| Enrofloxacin | 8 (6) | 3 (3) | 2 (2) |
| **Macrolides** |  |  |  |
| Erythromycin | 11 (8) | 7 (8) | 4 (4) |
| Azithromycin | 7 (5) | 4 (4) | - |
| Tylosin | 7 (5) | 2 (2) | 8 (9) |
| **Aminoglycosides** |  |  |  |
| Neomycin | 16 (12) | 9 (10) | 3 (3) |
| **Penicillins** |  |  |  |
| Amoxicillin | 21 (16) | 16 (18) | 40 (44) |
| **Polymyxins** |  |  |  |
| Colistin | 4 (3) | 8 (9) | 1 (1) |

**Table 4:** *Drugs, vaccines and associated products used within the 72 hours preceding visits to selected broiler (N=288), layer (N=288) and Sonali (N=192) farms in Bangladesh during January-May 2021.*

|  | **Broiler farms** | | **Layer farms** | | **Sonali farms** | |
| --- | --- | --- | --- | --- | --- | --- |
| **Product** | **Yes**  **n (%)** | **No**  **n (%)** | **Yes**  **n (%)** | **No**  **n (%)** | **Yes**  **n (%)** | **No**  **n (%)** |
| Vitamins | 218 (76) | 70 (24) | 217 (75) | 71 (25) | 186 (97) | 6 (3) |
| Minerals | 52 (18) | 236 (82) | 56 (19) | 232 (81) | 70 (36) | 122 (64) |
| Antibiotics | 135 (47) | 153 (53) | 107 (37) | 181 (63) | 102 (53) | 90 (47) |
| Antifungal | 43 (15) | 245 (85) | 60 (21) | 228 (79) | 65 (34) | 127 (66) |
| Antiprotozoal | 19 (7) | 269 (93) | 20 (7) | 268 (93) | 41 (21) | 151 (79) |
| Anthelminthic | 13 (5) | 275 (95) | 31 (11) | 257 (89) | 12 (6) | 180 (94) |
| Growth promoters | 17 (6) | 271 (94) | 14 (5) | 274 (95) | 24 (12) | 168 (88) |
| Probiotics | 82 (28) | 206 (72) | 95 (33) | 193 (67) | 75 (39) | 117 (61) |
| Vaccines | 69 (24) | 219 (76) | 52 (18) | 236 (82) | 66 (34) | 126 (66) |

**Table 5:** *Farm-level antibiotics used within the 14 days preceding visits to commercial broiler, layer and Sonali chicken farms in Bangladesh, during January-May 2021.*

|  | **Broiler (N=288)** | | **Layer (N=288)** | | **Sonali (N=192)** | |
| --- | --- | --- | --- | --- | --- | --- |
| **Variables** | **Number of farms**  **n (%)** | **95% CI** | **Number of farms**  **n (%)** | **95% CI** | **Number of farms**  **n (%)** | **95% CI** |
| **Usage of at least one antibiotic** | 282 (98) | 96-99 | 207 (72) | 66-77 | 169 (88) | 83-92 |
| **Purposes of antibiotic use** | **N=282** |  | **N=207** |  | **N=169** |  |
| Prophylaxis | 21 (8) | 5-11 | 34 (16) | 12-22 | 5 (3) | 1-7 |
| Treatment | 73 (26) | 21-31 | 45 (22) | 16-28 | 67 (40) | 32-47 |
| Both | 188 (67) | 61-72 | 128 (62) | 55-68 | 97 (57) | 50-65 |
| **Antibiotic suggested by** | **N=282** |  | **N=207** |  | **N=169** |  |
| Veterinary practitioner | 147 (52) | 46-58 | 120 (58) | 51-65 | 120 (71) | 64-78 |
| Pharmacy owner | 1 (1) | 1-2 | 3 (1) | 1-4 | - | - |
| Feed dealer | 102 (36) | 31-42 | 47 (23) | 17-29 | 43 (25) | 19-33 |
| Veterinary doctor from pharmaceutical company | 2 (1) | 1-3 | 27 (13) | 9-18 | 2 (1) | 1-4 |
| Quack | 1 (1) | 1-2 | - | - | - | - |
| Self-decision | 29 (10) | 7-14 | 10 (5) | 2-9 | 4 (2) | 1-4 |

**Table 6:** *Nature of interaction between farmers, feed dealers and other associated partners*

| **Characteristics** | **Broiler farms**  **n (%)** | **Layer farms**  **n (%)** | **Sonali farms**  **n (%)** |
| --- | --- | --- | --- |
| Presence of interaction between farmers and feed dealers | 229 (80) | 166 (58) | 149 (78) |
| **Types of support provided by feed dealers** |  |  |  |
| Feed supply to the farms | 225 (78) | 166 (58) | 142 (74) |
| Chick supply to the farms | 189 (66) | 116 (40) | 142 (74) |
| Medicine supply to the farms | 185 (64) | 137 (48) | 137 (71) |
| Selling chicken and chicken byproducts | 180 (63) | 87 (30) | 97 (51) |
| **Chicken production depends on** |  |  |  |
| Credits from feed dealers | 103 (36) | 63 (22) | 63 (33) |
| Credits from large commercial farms | 28 (10) | 29 (10) | 35 (18) |
| Credits from hatcheries | 27 (10) | 9 (3) | 31 (16) |
| Agreements between farmers and feed dealers | 106 (37) | 72 (25) | 78 (41) |
| Agreements between farmers and large farms | 2 (1) | 2 (1) | 2 (1) |
| Agreements between farmer and hatcheries | 8 (3) | 17 (6) | 6 (3) |
| No dependency (no financial agreements) | 73 (25) | 143 (50) | 47 (24) |

**Table 7:** *Comparison of antibiotic use in commercial chicken farms (N=768) by production type, flock size and areas, during January-May 2021, Bangladesh.*

| **Characteristics** | **Antibiotic use within last 24 hours** | | **OR, 95% CI** |
| --- | --- | --- | --- |
|  | **Yes** | **No** |  |
| **Chicken production type** |  |  |  |
| Layer | 90 | 198 | Ref. |
| Broiler | 134 | 154 | 1.91 (1.36-2.69) |
| Sonali | 90 | 102 | 1.94 (1.33-2.83) |
| **Flock size** |  |  |  |
| Small | 91 | 165 | Ref. |
| Medium | 112 | 144 | 1.41 (0.99-2.01) |
| Large | 111 | 145 | 1.39 (0.97-1.98) |
| **Areas for broiler and layer chicken** |  |  |  |
| Cumilla | 55 | 137 | Ref. |
| Gazipur | 103 | 89 | 2.88 (1.89-4.39) |
| Chattogram | 66 | 126 | 1.3 (0.85-2) |
| **Areas for Sonali** |  |  |  |
| Joypurhat | 30 | 66 | Ref. |
| Bogura | 60 | 36 | 3.67 (1.15-11.7) |

**Table 8:** *Factors associated with antibiotic use in commercial broiler farms (N=288), during January-May 2021, Bangladesh.*

| **Factors** | **Antibiotic use within last 24 hours** | | **OR, 95% CI** | ***p*** | **Adjusted OR,**  **95% CI** | ***P*** |
| --- | --- | --- | --- | --- | --- | --- |
|  | **Yes** | **No** |  |  |  |  |
| **Farm categories** |  |  |  |  |  |  |
| Small | 42 | 54 | Ref. |  |  |  |
| Medium | 46 | 50 | 1.18 (0.62-2.25) | 0.610 |  |  |
| Large | 46 | 50 | 1.18 (0.62-2.26) | 0.611 |  |  |
| **Study site** |  |  |  |  |  |  |
| Cumilla | 33 | 63 | Ref. |  | Ref. |  |
| Chattogram | 44 | 52 | 1.61 (0.5-5.22) | 0.423 | 1.12 (0.55-2.29) | 0.754 |
| Gazipur | 57 | 39 | 2.79 (0.93-8.34) | 0.066 | 2.33 (1.02-5.33) | 0.044 |
| **Age of the broiler chicken flock** | |  |  |  |  |  |
| ≥31 days | 5 | 11 | Ref. |  |  |  |
| 16-30 days | 103 | 118 | 1.92 | 0.367 |  |  |
| 1-15 days | 26 | 25 | 2.29 | 0.303 |  |  |
| **Presence of current illnesses in the chicken flock** | | | |  |  |  |
| No | 40 | 145 | Ref. |  | Ref. |  |
| Yes | 94 | 9 | 37.86 (13.32-107.64) | <0.001 | 41.22 (13.63-124.62) | < 0.001 |
| **Received training on chicken production** | | | |  |  |  |
| Yes | 30 | 58 | Ref. |  |  |  |
| No | 104 | 96 | 2.09 (1.12-3.91) | 0.02 |  |  |
| **Knowledge on the purpose of antibiotic use** | |  |  |  |  |  |
| Used to treat viral diseases | 5 | 12 | Ref. |  | Ref. |  |
| Used to treat bacterial diseases | 9 | 15 | 1.44 (0.49-4.62) | 0.540 | 2.73 (1.23-6.06) | 0.013 |
| Used to treat all diseases | 113 | 121 | 2.24 (0.78-6.43) | 0.133 | 3.25 (1.69-6.26) | <0.001 |
| Used to increase production | 7 | 6 | 2.8 (0.95-8.25) | 0.062 | 6.69 (2.8-16) | <0.001 |
| **Farmers education** |  |  |  |  |  |  |
| Graduate | 17 | 26 | Ref. |  |  |  |
| Higher Secondary | 25 | 24 | 1.59 (0.82-3.09) | 0.168 |  |  |
| Secondary | 67 | 70 | 1.46 (0.74-2.89) | 0.273 |  |  |
| Primary | 20 | 31 | 0.99 (0.55-1.75) | 0.964 |  |  |
| Illiterate | 5 | 3 | 2.55 (0.48-13.5) | 0.271 |  |  |
| **Farming experiences** | |  |  |  |  |  |
| > 10 years | 41 | 50 | Ref. |  |  |  |
| 5-10 years | 31 | 53 | 0.71 (0.42-1.2) | 0.207 |  |  |
| 1-5 years | 47 | 37 | 1.55 (0.94-2.56) | 0.088 |  |  |
| < 1 year | 14 | 14 | 1.22 (0.45-3.27) | 0.694 |  |  |
| **Heard of AMR** |  |  |  |  |  |  |
| Yes | 78 | 107 | Ref. |  |  |  |
| No | 56 | 47 | 1.63 (0.95-2.81) | 0.076 |  |  |
| **Antibiotic suggested by** | |  |  |  |  |  |
| Veterinary doctor | 66 | 80 | Ref. |  |  |  |
| Feed dealer | 61 | 61 | 1.21 (0.81-1.82) | 0.353 |  |  |
| Farmer | 7 | 13 | 0.65 (0.29-1.45) | 0.297 |  |  |

Model fit: model χ2 20.81, *p* 0.06 and R^2^0.388

**Table 9:** *Factors associated with antibiotic use in commercial layer farms (N=288), during January-May 2021, Bangladesh.*

| **Factors** | **Antibiotic use within last 24 hours** | | **OR, 95% CI** | ***p*** | **Adjusted OR,**  **95% CI** | ***p*** |
| --- | --- | --- | --- | --- | --- | --- |
|  | **Yes** | **No** |  |  |  |  |
| **Farm categories** |  |  |  |  |  |  |
| Small | 23 | 73 | Ref. |  |  |  |
| Medium | 32 | 64 | 1.58 (0.91-2.75) | 0.101 |  |  |
| Large | 35 | 61 | 1.82 (0.86-3.85) | 0.117 |  |  |
| **Study site** |  |  |  |  |  |  |
| Cumilla | 22 | 74 | Ref. |  | Ref. |  |
| Chattogram | 22 | 74 | 1 (0.32-3.1) | 1 | 1.08 (0.44-2.66) | 0.624 |
| Gazipur | 46 | 50 | 3.09 (1.05-9.08) | 0.040 | 3.79 (1.71-8.38) | <0.001 |
| **Age of the layer chicken flock** | |  |  |  |  |  |
| ≥181 days | 62 | 155 | Ref. |  | Ref. |  |
| 91-180 days | 15 | 26 | 1.44 (0.69-3.01) |  | 0.99 (0.45-2.24) |  |
| 31-90 days | 9 | 11 | 2.04 (0.84-4.98) |  | 2.98 (1.45-6.11) |  |
| 16-30 days | 4 | 5 | 2 (0.53-7.51) |  | 1.66 (0.34-8.16) |  |
| 1-15 days | 0 | 1 | undefined |  | undefined |  |
| **Presence of current illnesses in the chicken flock** | | |  | |  |  |
| No | 40 | 190 | Ref. |  | Ref. |  |
| Yes | 50 | 8 | 29.68 (9.38-93.92) | <0.001 | 36.45 (9.52-139.43) | <0.001 |
| **Received training on chicken production** | | |  | |  |  |
| Yes | 31 | 60 | Ref. |  |  |  |
| No | 59 | 138 | 0.82 (0.43-1.57) | 0.564 |  |  |
| **Knowledge on the purpose of antibiotic use** | |  |  |  |  |  |
| Used to treat viral diseases | 4 | 10 | Ref. |  |  |  |
| Used to treat bacterial diseases | 9 | 38 | 0.59 (0.27-1.28) | 0.183 |  |  |
| Used to treat all diseases | 71 | 145 | 1.22 (0.39-3.85) | 0.730 |  |  |
| Used to increase production | 6 | 5 | 3 (1.03-8.78) | 0.045 |  |  |
| **Farmers education** |  |  |  |  |  |  |
| Graduate | 12 | 41 | Ref. |  |  |  |
| Higher Secondary | 22 | 24 | 2.21 (0.88-5.56) | 0.092 |  |  |
| Secondary | 48 | 107 | 1..53 (0.87-2.69) | 0.136 |  |  |
| Primary | 8 | 16 | 1.7 (0.6-4.84) | 0.314 |  |  |
| **Farming experiences** | |  |  |  |  |  |
| > 10 years | 50 | 70 | Ref. |  |  |  |
| 5-10 years | 20 | 64 | 0.44 (0.23-0.83) | 0.011 |  |  |
| 1-5 years | 17 | 57 | 0.42 (0.18-0.97) | 0.043 |  |  |
| < 1 year | 3 | 7 | 0.6 (0.24-1.5) | 0.275 |  |  |
| **Heard of AMR** |  |  |  |  |  |  |
| Yes | 54 | 141 | Ref. |  |  |  |
| No | 36 | 57 | 1.65 (0.75-3.62) | 0.212 |  |  |
| **Antibiotic suggested by** | |  |  |  |  |  |
| Veterinary doctor | 66 | 142 | Ref. |  |  |  |
| Feed dealer | 20 | 49 | 0.88 (0.32-2.41) | 0.801 |  |  |
| Farmer | 4 | 7 | 1.23 (0.21-7.06) | 0.817 |  |  |

Model fit: model χ2 10.67, *p* 0.6387 and R^2^0.3308

**Table 10:** *Factors associated with antibiotic use in commercial Sonali farms (N=192), during January-May 2021, Bangladesh*

| **Factors** | **Antibiotic use within last 24 hours** | | **OR, 95% CI** | ***p*** | **Adjusted OR, 95% CI** | ***P*** |
| --- | --- | --- | --- | --- | --- | --- |
|  | **Yes** | **No** |  |  |  |  |
| **Farm categories** |  |  |  |  |  |  |
| Small | 26 | 38 | Ref. |  |  |  |
| Medium | 34 | 30 | 1.66 (0.75-3.64) | 0.209 |  |  |
| Large | 30 | 34 | 1.29 (0.73-2.28) | 0.383 |  |  |
| **Study site** |  |  |  |  |  |  |
| Joypurhat | 30 | 66 | Ref. |  |  |  |
| Bogura | 60 | 36 | 3.67 (1.15-11.7) | 0.028 |  |  |
| **Age of the Sonali chicken flock** | | |  |  |  |  |
| ≥181 days | 5 | 26 | Ref. |  | Ref. |  |
| 91-180 days | 7 | 18 | 2.02 (0.61-6.71) |  | 3.67 (1.72-7.82) | 0.001 |
| 31-90 days | 31 | 34 | 4.74 (1.62-13.85) |  | 7.09 (2.43-20.67) | <0.001 |
| 16-30 days | 41 | 22 | 9.69 (3.29-28.57) |  | 12.76 (2.59-62.75) | 0.002 |
| 1-15 days | 6 | 2 | 15.6 (5.26-46.23) |  | 34.75 (11.85-101.86) | <0.001 |
| **Presence of current illnesses in the chicken flock** | | | |  |  |  |
| No | 29 | 94 | Ref. |  | Ref |  |
| Yes | 61 | 8 | 24.72 (5.59-109.3) | <0.001 | 28.47 (4.97-162.97) | <0.001 |
| **Received training on chicken production** | |  |  |  |  |  |
| Yes | 6 | 12 | Ref. |  |  |  |
| No | 84 | 90 | 1.87 (0.77-4.53) | 0.168 |  |  |
| **Knowledge on the purpose of antibiotic use** | | |  |  |  |  |
| Used to treat viral diseases | 10 | 23 | Ref. |  |  |  |
| Used to treat bacterial diseases | 29 | 42 | 1.59 (0.8-3.14) | 0.183 |  |  |
| Used to treat all diseases | 50 | 36 | 3.19 (0.79-12.91) | 0.103 |  |  |
| Used to increase production | 1 | 1 | 2.3 (0.89-5.87) | 0.082 |  |  |
| **Farmers education** |  |  |  |  |  |  |
| Graduate | 9 | 17 | Ref. |  |  |  |
| Higher Secondary | 8 | 17 | 0.88 (0.34-2.35) | 0.812 |  |  |
| Secondary | 44 | 44 | 1.89 (0.73-4.89) | 0.19 |  |  |
| Primary | 23 | 21 | 2.07 (0.88-4.84) | 0.094 |  |  |
| Illiterate | 6 | 3 | 3.78 (0.23-61.6) | 0.351 |  |  |
| **Farming experiences** |  |  |  |  |  |  |
| > 10 years | 26 | 47 | Ref. |  |  |  |
| 5-10 years | 23 | 25 | 1.66 (0.74-3.75) | 0.22 |  |  |
| 1-5 years | 41 | 30 | 2.47 (1.31-4.67) | 0.005 |  |  |
| **Heard of AMR** |  |  |  |  |  |  |
| Yes | 45 | 68 | Ref. |  |  |  |
| No | 45 | 34 | 2 (0.77-5.22) | 0.157 |  |  |
| **Antibiotic suggested by** |  |  |  |  |  |  |
| Veterinary doctor | 61 | 71 | Ref. |  |  |  |
| Feed dealer | 27 | 27 | 1.16 (0.47-2.86) | 0.741 |  |  |
| Farmer | 2 | 4 | 0.58 (0.19-1.76) | 0.338 |  |  |

Model fit: model χ2 6.86, *p* 0.1432and R^2^0.3835

**Figure 1:** Map of the study sites for commercial chicken farms sampling
