# Supplementary material for: Antibiotic usage practices and its drivers in commercial chicken production in Bangladesh
Source: PLoS One. 2022 Oct 17;17(10):e0276158. doi: 10.1371/journal.pone.0276158 (PMC9576089; doi:10.1371/journal.pone.0276158)
Supplement: Attachment — Submitted filename: PONE-D-22-06825_Reviewers comments_responses.docx [file pone.0276158.s005.docx]

**PLOS ONE Editorial Board Comments to the Author:** First and most important is that you give a clear explanation what is different from the former studies you made on the subject. As one of the reviewers noted this publication seems to be the same as 2 former studies. Publishing the same manuscript in different journals cannot be accepted, and will automatically lead to the rejection of the manuscript if not clear difference between this manuscript and the former manuscripts are given. Second the statistical methods used are not adequate and are erroneous so they need through adaptation. There are thus major issues with the manuscript.

***Response:*** *Thank you for sharing the reviewers’ helpful comments and suggestions. Under this study, we surveyed and collected data from a large number of commercial chicken farms (n=768) in different chicken production types (broiler, layer, and Sonali chicken) and scales (small, medium and large farm). Antibiotic usage data was collected over different timeframes (24 hours, 72 hours, 14 days preceding our farm visit, and over the production cycle). The sample size for previously published studies was smaller than our study. The earlier studies were confined to single production (broiler or layer) and limited to small geographical locations. The antibiotic usage reported by previous studies was for the whole production period. The antibiotic usage prevalence for the whole production period is less useful measure to assess the problem properly associated with antibiotic usage in the commercial chicken production. Most importantly, the antibiotic usage prevalence reported by our study was estimated over different timeframes (point prevalence) that would be more useful to the policymakers, animal health practitioners, farmers, and animal feed dealers for providing interventions in reducing unnecessary use of antibiotics.*

*Detail responses to each comment are given below for your consideration.*

**Reviewer: 1**

**Comment:** I would suggest using “antimicrobial” instead of “antibiotic” (for the entire manuscript).

***Response:*** *Thanks for your suggestion. Antimicrobial is the broader term that includes antibiotics, antiviral, antifungals and antiparasitic. Under this study, we mainly collected information about antibiotic usage which is related with AMR (antimicrobial resistance). Therefore, we would prefer to use “antibiotic” instead of antimicrobials.*

**Comment:** Please specify “antibiotic usage” (are you presenting data about quantification, practices, surveillance, etc.?).

Your title should be more specific/detailed.

***Response:*** *Thanks for your suggestion. We have added a word “practice” in the title (line number 1). The study covered wider geographical regions of Bangladesh. Chicken farms were selected from five districts; Gazipur, Chattogram, Cumilla, Joypurhat and Bogura. We chose the areas because the majority of chicken farms are located in these areas. The findings of the study represent the real picture of antibiotic usage in commercial chicken in Bangladesh.*

**Comment:** Is it a mean? If yes, provide a 95% CI.

If 23% if for a type of production and 31% another type of production, then they should be presented separately.

***Response:*** *Thanks for your suggestion. We can’t add more texts in the abstract section because of word limit instructed by journal. Detail information are given in the main body of the manuscript. (Table 3, page number 17).*

**Comment:** Use antimicrobial everywhere else in the manuscript.

***Response:*** *Thanks. We have revised accordingly.*

**Comment:** In general, references are not always relevant or appropriate. Please review.

***Response:*** *Thanks for your suggestions. Very useful. We have added updated references in the revised manuscript.*

**Comment:** You can use the WHO or OIE action plans on Amu-AMR for example.

***Response:*** *Thanks. We have added your suggested reference (line number 50).*

**Comment:** You reference is for aquaculture. Could you find another one in avian production?

***Response:*** *Thanks. We have added more relevant references in the revised manuscript (line number 52-53).*

**Comment:** This statement is not exactly true.

Quantification of AMU is now performed in a lot of countries. See the Fifth OIE Annual Report on Antimicrobial Agents Intended for Use in Animals.

***Response:*** *We agree with you. We have removed the wrong statement from the revised manuscript (line number 54).*

**Comment:** Reference 5 is related to pigs only and reference is from 2012 – please provide more up-to-date information.

***Response:*** *Thanks. We have removed the old reference and added updated reference in the revised manuscript (line number 55).*

**Comment:** A 140-farm study is not considered a small-scale study.

Please provide a summary of main findings from this important study in your introduction.

*Response: Thanks for your comment. We have added more information in the revised manuscript (line numbers 67-68).*

**Comment:** Could you give more details please? Antimicrobials cannot be introduced into feed during manufacturing, but can antimicrobials be added/mixed to the feed later in the process (on the farm, by the farmer himself, for example)?

***Response:*** *Thanks for your suggestions. Farmers usually administered antimicrobials to the chicken through water and feed. But nothing is mentioned in the law about your proposed statement ‘can antimicrobials be added/mixed to the feed later in the process’. We have added more information about this in the revised manuscript (line numbers 82-83).*

**Comment:** Please give more information in your introduction about the conclusions of other important studies conducted recently in Bangladesh (references 7, 9, 11, 12, 13, and 14) and why your study is essential in this context. What question exactly are you trying to answer with this study? How different your study is compared to the other ones, especially ref 9 (Imam et al. 2020), ref 12 (Kalam et al.2021), and ref 13 (Islam et al. 2016)?

***Response:*** *Thanks for your valuable comments and suggestions. We have revised the section considering your suggestion (line numbers 63-69).*

**Comment:** Please use  [**https://doi.org/10.1111/zph.12315**](https://doi.org/10.1111/zph.12315)

to report all important information related to your study design (STROBE Statement – Checklist of items that would be included in reports of cross-sectional studies).

***Response:*** *Thanks for your suggestion. We have included additional information considering STROBE statement.*

**Comment:** Explain why you recruited from 3 districts for broilers and layers, and only from 2 districts for Sonali farms. And provide more details on how different those areas in Bangladesh are similar or different from other areas not included in your study.

***Response:*** *We choose Gazipur, Chattogram, and Cumilla districts because of higher broiler and layer farm density compared to other districts in Bangladesh. Similarly, we choose Joypurhat and Bogura districts because of higher Sonali chicken farm density. We have added more information in the revised manuscript (line numbers 113-116).*

**Comment:** Provide the source population (how many farms in the upazilas?) and the target population.

***Response:*** *Limited time and funding did not allow us to conduct a census of all farms in each selected upazila to support their random sampling. Total 288 broiler and 288 layer farms were enrolled in 12 upazilas in 3 districts, and 192 Sonali farms were enrolled in 8 upazilas in 2 districts. We included this information in line numbers 127-128.*

**Comment:** non randomly recruited – please specify

Discuss the bias

***Response:*** *Yes. We used purposive sampling instead of random sampling to select farms in each sampling stratum.* *Limited time and funding did not allow us to conduct a census of all farms in each selected upazila to support their random sampling. We have mentioned this information in the limitation section (line numbers 391-394).*

**Comment:** EXPLAIN how you decided 32% (based on a specific reference or preliminary study?)

Did you expect the same prevalence in the 3 types of farms?

Please give the calculation of sample size (what was the estimated for your sample size?)

***Response:*** *Based on the previous study findings, we expected to estimate 32% prevalence of antibiotic usage in commercial chicken farms. The required number of sample size was 768 assuming a 32% expected prevalence, with 95% level of significance and 5% precision. We have included the reference (16) as a source of information for 32% prevalence (line numbers 129-130).*

**Comment:** One in each upazila?

Did you have access to an exhaustive list of all feed dealers in the regions?

How did you recruit them exactly for the interview?

***Response:*** *Limited time and funding did not allow us to conduct a census of all feed dealers. We interviewed total 20 large feed dealer purposively selected who used to sell larger amount of poultry feed and medicine.*

**Comment:** Provide the interview details (how many questions? Ideally provide the questionnaire).

***Response:*** *Thanks for your suggestion. Two questionnaires (farmers and feed dealers) have been attached as supplementary files with the main manuscript (line number 147 and 156).*

**Comment:** Provide ethic committee approval number if you got an approval

***Response:*** *The study protocol was reviewed and approved by icddr,b Research Review Committee, Ethical Review Committee and Animal Experimentation Ethics Committee (PR-20116). We have included this information under Ethical Approval section (line number 424).*

**Comment:** Please provide both questionnaire as a supplemental information. Not clear how exactly you collected data. Type of questions (open, closed, type of variable for the analyses) and how each answer was used in the analyses.

Example: “farm demographics” could be related to the number of chickens at the time of the visit or to the number of chickens reared each year or to the number of facilities on the farm or a lot of different other data! Please specify the type of data for each variable.

***Response:*** *Two detail questionnaires (farmers and feed dealers) have been attached as supplementary files with the main manuscript (line number 147 and 156).*

**Comment:** Please provide details. Mean + 95%CI, median, range?

***Response:*** *Thanks for your suggestion. We have added frequency, mean, standard deviation, p-value and 95% confidence interval in the revised manuscript (line numbers 162-163).*

**Comment:** Specify what variables

***Response:*** *We have attached supplementary files that allow readers to see all variables at a glance.*

**Comment:** Please provide one reference to support your statistical decisions.

***Response:*** *Thanks for your suggestion. We have added a reference in the revised manuscript (line number 177).*

**Comment:** Provide the average number of chickens in each category (small, medium, large farms for broiler, layer, and Sonali farms recruited).

***Response:*** *Thanks. We have added your suggested information in the revised manuscript (line numbers 188-190).*

**Comment:** What is this range? Is 88% related to one type of production and 91% another type of production? If yes, then present the proportions separately for each production (otherwise, provide a mean for the 768 farms with a 95% CI).

Same question for all ranges presented. Please specify. Maybe use a table to present those results.

***Response:*** *Among the 768 surveyed commercial chicken farms,* *688 farms (90%, 95% CI: 87-92%) only raised one production type of chicken (either broiler or layer or Sonali), and 9-12% of farms raised different types of chicken production.*

**Comment:** Provide %

***Response:*** *Included.*

**Comment:** Provide 95% CI each time you present a mean

***Response:*** *Included (line numbers 195-196).*

**Comment:** Provide 95% CI – maybe you have overlap of the CI and it is then not statistically higher.

***Response:*** *Included in the revised manuscript (line number 201).*

**Comment:** Same here – add statistics (is it statistically significant?)

***Response:*** *Included in the revised manuscript (line number 208-209).*

**Comment:** In this case, it is no longer “prescribed” – change the verb in this sentence for maybe “recommended” or “decided” or change the structure of this sentence.

***Response:*** *Thanks for your suggestion. We have replace the term “prescribed” by “recommended” (line number 214).*

**Comment:** This result depends on the duration (number of days) between the starting date of the production cycle and the date of visit. Please provide more information to highlight the results in relation to this confounding factor.

***Response:*** *Totally agree with you. We have revised the section considering your suggestion (line numbers 220-221).*

**Comment:** Did you expect a difference? Please explain.

***Response:*** *We expected a difference for the purposes of antibiotic use because of age and production types. By this study, we found that the practices were similar in different point time frame.*

**Comment:** Same question here: why did you ask the same question with 24 hours versus 14 days (what difference did you expect?)?

***Response:*** *Thanks. Explained in previous comment.*

**Comment:** Meaning that all chicks were sick on day 1 and treated because of clinical signs observed at the arrival on the farm?

I am wondering if all farmers knew the difference between prophylaxis and treatment in your study. Did you define the terms with them at the beginning of the interview? If yes, provide the definition that was provided.

***Response:*** *Thanks for sharing your concern. I have rechecked the dataset. The correct information would be “55% broiler farms, 42% layer farms and 21% Sonali farms used antibiotics on the first day of the batch production cycle prophylactically”. We have included the correct information in the revised manuscript (line numbers 235-236).*

**Comment:** About the routes of administration, it is not completely clear. Please provide all routes of administration with their frequency of use.

***Response:*** *Most of the broiler (99%), layer (92%) and Sonali (100%) chicken farmers administered antibiotic to chicken through water. Added more information about this in the revised manuscript (line numbers 248-249).*

**Comment:** Do you have information on the diseases diagnosed on the farms?

***Response:*** *We did not collect this information.*

**Comment:** Please provide information on data collection in the M&M (did the research team go into the farm to observe the actual number of sick animals?).

***Response:*** *According to the farmers’ report, the overall proportion of sick chickens per farm within the last 14 days preceding our farm visit was 71 per 1000 broiler, 21 per 1000 layer and 31 per 1000 Sonali farms, whereas the overall proportion of dead chickens was 39 per 1000 broiler, 9 per 1000 layer and 23 per 1000 Sonali chickens. We have clarified the timeframe in the revised manuscript (line numbers 256-257).*

**Comment:** This type of information was known from the beginning of the study and should be provided in the introduction (context) or in the M&M. It was known in order to build your questionnaire from my understanding.

***Response:*** *Generally, we know this but our study generated specific evidence on this issue. That’s why we included this information in the result section.*

**Comment:** In your results section, please clearly divide between the results obtained from the farmers versus the results obtained from the feed dealers.

***Response:*** *Thanks for your suggestions. We have tried to explain the data separately.*

**Comment:** Confounding factor here! Maybe there are many more broiler farms than other types of production… Not sure about how to interpret this result. Misleading conclusion.

***Response:*** *According to our observation during feed dealers visit, broiler farmers (42%) mostly came to bought medicine followed by Sonali (40%) and layer (18%) farmers. In the method section, we have clearly mentioned the data collection procedure. During each feed dealer visit, drug dispensing data was collected from five consecutive chicken farmers. Descriptive statistics were only presented for feed dealers’ data. So, confounding factor will not be a matter of concern.*

**Comment:** Did you verify that they actually knew? Did you ask them to provide an explanation of what they think AMR is?

***Response:*** *No further information was collected to verify.*

**Comment:** Not statistically significant… not true as stated. OR contains 1.

***Response:*** *Yes. You are right. In bivariable analysis, we did not find the significant association for the variable.*

**Comment:** You found a difference btw areas (Gazipur had a significant higher usage than Cumilla and Chattogram for example).

***Response:*** *Yes.*

**Comment:** Not sure how to interpret this final sentence – please explain what this means exactly.

***Response:*** *These findings were used to explain variance and measure goodness-of-fit for multivariate regression model that we built here.*

**Comment:** Please inform about the external validity of the study (ability to extrapolate results beyond the source population) and internal validity (ability to extrapolate results from the study group to the source population).

Please discuss BIASES in your study and confounding factors because you have a lot of them (mainly related to the type of sampling).

***Response:*** *The study sites and study population that we used in this study represent the real picture of Bangladesh as these areas are considered as hub for broiler, layer and Sonali chicken. We adjusted confounding variables and checked collinearity in the final multivariate logistic model. The study has some limitations that we mentioned in the manuscript (line numbers 391-397).*

**Comment:** What about the ranking of those antimicrobials by the World Health Organization? (use the 6th revision of the WHO ranking of antimicrobials)

Please provide information on the Highest Priority Critically Important Antimicrobials used in your study.

***Response:*** *Thanks for your suggestion. The categorization of antimicrobials by OIE and WHO was not similar. According to WHO, antimicrobials are categorized into three groups; access, watch and reserve group. As our manuscript focusing animals, we mentioned OIE guideline instead of WHO. Colistin is recognized as category of Highest Priority Critically Important Antimicrobials by WHO. We have added this information in the revised manuscript (line numbers 332-335).*

**Comment:** Not statistically significant and not sure that the fact that a farmer answered “yes” to the question “Heard of AMR” is equivalent to “understanding bout AMR”. Please discuss the limits and biases of your study.

***Response:*** *Thanks for your comment. Concurrent chicken morbidity and farm location were the only identified factors that were significantly associated with increased antibiotic usage in commercial chickens. We have modified the section in the revised manuscript (line number 370).*

**Comment:** You should keep the word “prescription” only to veterinarians or doctors (prescribers). Providers of antimicrobials are not necessarily prescribers.

***Response:*** *Thanks for your suggestion. We agree with you. We have used “recommended” instead of prescription in the revised manuscript (line number 389).*

**Comment:** This is a huge bias in your study and you should explain it better in your discussion.

***Response:*** *Thanks for your comment. This is very difficult to perform random sampling from upazilas where thousands of chicken farms are located using limited resources. This may not have influenced the study findings as the farm characteristics and reported antimicrobial usage were consistent with an earlier study. The stratified sampling that we used in this study was also useful to minimize the sampling bias in some extent.*

**Comment:** Provide information on the type of support (financial? Provide the details)

***Response:*** *The statement is recommended by icddr,b Board of Trustee. So, I can’t change the statement.*

**Comment:** Please consider providing the data in supplementary material for transparency.

***Response:*** *Thanks for your suggestion. I will include the data.*

**Comment:** Explain the significance of the % (it seems that it is related to the type of production (total of 100% for a specific type of production and type of characteristic evaluated).

Please consider adding a row with the total (independent of the area) especially if you don’t expect differences related to the area

I am wondering about the significance of the % with the stratified sampling that you used. For example, 2% of broiler farms were medium farms and had 2 batches of chickens at the time of the visit. What is the relevance of this information?

Maybe consider having the calculation of % only by row and by column? For example, 100%, 95%, and 82% of small, medium, and large broiler farms had only one batch of chickens observed during a single visit.

Please provide a 95% CI.

***Response:*** *Thanks for your comments and suggestions. Regarding your comments about* the significance of the % with the stratified sampling, 92% of broiler farms had single batch during our visit and 7% of farms had two batches. The statistics that we gave is useful calculate total number. *The table has lots of information. That’s why we did not include 95% CI. I think that p value provides meaningful information that are useful to the readers. We have added more information in the revised manuscript considering your suggestions (page number 16, Table 1).*

**Comment:** Did you always get only one answer for this question? It seems surprising that you had no one answering “part is from my veterinarian and part is from the feed dealer or my own decision”.

***Response:*** *Thanks. This was not a general question. It was specific for antibiotic use within the 24 hours preceding visits. So, farmers’ response was either veterinarian or feed dealer or own decision or other.*

**Comment:** Could you define this n=1 answer?

Did you ask “Who suggested the antibiotic?” and the farmer really answered “a quack”? How did you interpret this answer?

***Response:*** *Among the 134 farmers who used antibiotics within 24 hours, one farmer responded that he got suggestion from a quack.*

**Comment:** In your tables in general, it is not clear what is your denominator for the calculation of the %. Please continue to provide a 95% CI.

***Response:*** *We have mentioned the denominator in the title of table [broiler (N=134), layer (N=90) and Sonali (N=90)]. We have added 95% CI in the revised manuscript (page number 18, Table 3)*

**Comment:** What is the relevance of each information? For example, vaccination may not have been used in the 72 hours preceding the visit but still be up to date… What can you conclude from the information that 69/288 broiler farmers administered a vaccine in the last 72 hours?

***Response:*** *The information other than antibiotics are not directly relevant to our study objectives. As we collected other medicine use history, we included this information in the manuscript. This information may be useful for future studies.*

**Comment:** Could you confirm that “selling chicken and chicken byproducts” is from the farmer to the feed dealer? Does it mean that the farmer sells chickens and eggs to the feed dealer? In this case, is it truly a "support provided by feed dealer”? Did you have “byproducts” sold other than eggs? If no, then I would write “Selling chicken and eggs”

***Response:*** *Thanks for your suggestion.* Selling chicken and eggs is appropriate. We have modified the statement in the revised manuscript (page number 20, Table 6)

**Comment:** No need to present the p value because you already present the OR with its 95% CI

**Response:** I totally agree with you. However, I am interested to include p value as some readers may find this information useful.

**Comment:** Explain and give details in the text.

**Response:** *We have added more information in the method and result section.*

**Comment:** Please provide at least the name of the districts on this map.

**Response:** *Thanks for your suggestion. We have included district name in the map.*

**Reviewer: 2**

**Comment:** Contribute instead of contributed

***Response:*** *Revised accordingly.*

**Comment:** So antibiotic usage was estimated based on structured interviews? This could be described more clearly here.

***Response:*** *Yes. Structured interviews were conducted with 288 broiler, 288 layer and 192 Sonali (locally-produced cross-bred) farmers in 20 sub-districts across Bangladesh. We can’t add more detail about the interview process, farm selection and questionnaires because of word limit.*

**Comment:** add "risk factors

***Response:*** *Thanks. Added “risk factors”.*

**Comment:** I would say estimations from experts. Also, the referenced article is not the original research. In the paper referred to here two other studies are referred to: "The amount of antibiotics consumed by livestock worldwide is almost double that used by humans, according to some estimates1,2"

***Response:*** *Thanks for your comment and suggestion. We have revisited the article and searched more article for updated information. We have revised the sentence based on updated information from a article (Van Boeckel, T.P., et al., Global trends in antimicrobial resistance in animals in low-and middle-income countries. Science, 2019. 365) (line numbers 54-55).*

**Comment:** add one sentence providing the system they use and whether the slaughter age of the chicken used for meat is the same at the age of the ones who lay eggs

***Response:*** *Thanks for your suggestion. We have added a sentence in the revised manuscript (line number 63-64).*

**Comment:** As far as I understand the study was carried out form Jan - May, however, the information in commercial farms was gathered at one single moment, referring to the previous period. To make this clear I would start the sentence with the time frame, to make clear that the he data have not been collected from Jan to May 2020

***Response:*** *Thanks for your suggestion. Over the study period, every selected farm was visited once to collect data. We have added this information in the revised manuscript (line number*

*105-106).*

**Comment:** Suggestion to provide a formula here. The 95% mentioned here is the level of confidence I think.

***Response:*** *Thanks for your suggestion. We have corrected the sentence considering your suggestion.*

**Comment:** What data was collected on antibiotic usage?

***Response:*** *We asked farmers whether they administered antibiotics to their chicken or no. So, the response was either “yes” or “no” at different point of time (24 hours, 72 hours and 14 days).*

**Comment:** This suggests that the level of antibiotic usage was measured, while only the frequency of usage was estimated right?

***Response:*** *Yes. We measured the frequency of antibiotic usage at different point of time.*

**Comment:** How is "inappropriate knowledge" determined?

***Response:*** *We asked a question to the farmers about the Knowledge on the purpose of antibiotic use. Based on their response, we have defined it “inappropriate”. The majority of the broiler farmers (n=234, 81%), layer farmers (n=216, 75%) and Sonali farmers (n=86, 45%) said that they used antibiotics to treat all diseases. We presented detail statistics in table 8, 9, 10.*

**Comment:** The timeframes used here might have quite a big impact on the frequency of use, as it can be expected that antibiotics are used most frequently at the start of the production cycle.

***Response:*** *Yes. We agree with you. According to the farmers’ report, 55% broiler farms, 42% layer farms and 21% Sonali farms used antibiotics on the first day of the batch production cycle prophylactically. We have added this information in the revised manuscript (line number 234-235).*

**Comment:** What do these percentages mean? Estimated percentage of farms with use of CIAs?

***Response:*** *Yes. You are right. We have clarified the sentence in the revised manuscript considering your suggestion (line number 344).*

**Comment:** This was not shown I think. It was shown that farms in these areas used antibiotics more frequently. The level of usage was not measured.

***Response:*** *We agree with you. Farms located in Gazipur and Bogura used antibiotics frequently than in other areas. We have modified the sentence in the revised manuscript (line number 379).*

**Comment:** please mentioned if you have seen any seasonal effects between January and May that could have influenced your results

***Response:*** *We did not find any significant variation of antibiotic usage due to seasonal variation. Therefore, we have mentioned this in the manuscript.*

**Comment:** As a step in between regular surveys might also be an option?

***Response:*** *Thanks for your suggestion. We have used “regular” instead of “continuous” monitoring in the revised manuscript (line number 405).*
